# Burden of common infectious diseases in children with growth failure from 1990 to 2021: analysis of the Global Burden of Disease Study

**DOI:** 10.3389/fped.2025.1648964

**Published:** 2025-11-07

**Authors:** Yujie Li, Jin Xu, Zhaoqi Li, Meng Ren, Shanping Jiang

**Affiliations:** 1 Department of Pulmonary and Critical Care Medicine, Sun Yat-sen Memorial Hospital, Sun Yat-sen University, Guangzhou, China; 2 Institute of Pulmonary Diseases, Sun Yat-sen University, Guangzhou, China; 3 Department of Endocrinology, Sun Yat-Sen Memorial Hospital, Sun Yat-sen University, Guangzhou, China; 4 Guangdong Clinical Research Center for Metabolic Diseases, Guangzhou Key Laboratory for Metabolic Diseases, Guangzhou, China

**Keywords:** GBD, global, child growth failure, diarrheal diseases, malaria, measles, respiratory infections and tuberculosis

## Abstract

**Background:**

Child undernutrition is a serious public health problem that is associated with various infectious diseases (diarrheal diseases, malaria, measles, respiratory infections and tuberculosis). However, an assessment of the global burden of common infectious diseases in children with growth failure is still needed.

**Objective:**

This study aims to quantify the global burden of common infectious diseases in children with growth failure and to project future trends to 2050 to contribute to public health interventions.

**Methods:**

By analyzing the Global Burden of Disease Study 2021 (GBD 2021), we assessed the correlation and trend of common infectious diseases in malnourished children, stratified by age, country, and territory. In addition, Bayesian age–period–cohort (BAPC) models were used to predict future patterns until 2050.

**Results:**

We found that undernutrition among children is closely associated with common infectious diseases. The rate of deaths and disability-adjusted life years among children with diarrheal diseases, malaria, and respiratory infections and tuberculosis was negatively correlated with age. The prevalence of measles was highest in 1–2-year-olds. Diarrheal diseases and malaria are prevalent in Nigeria, measles is endemic in Somalia, and respiratory infections and tuberculosis are widespread in Nigeria and India. The BAPC results show that the malaria burden may increase in the future.

**Conclusion:**

This study emphasizes the burden of common infectious diseases in children with growth failure and facilitates international aid and WHO decision-making targeting countries and age groups.

## 1 Introduction

Child undernutrition is a major public health problem that leads to a variety of dangerous and complex complications. Approximately 45% of deaths among children under 5 years old are associated with undernutrition (1), a condition termed child growth failure. This includes child stunting, child underweight, and child wasting, defined as height-for-age, weight-for-age, and weight-for-height being ≤2 standard deviations below the WHO child growth standards median. According to data from the Global Burden of Disease Study 2016 (GBD 2016), among children under 5 years old in sub-Saharan Africa, 36.6% were stunted, 8.6% were underweight, and 19.5% were wasted (2). Furthermore, children suffering from undernutrition are more vulnerable to infectious diseases, including gastroenteritis, malaria, pneumonia, and so on, which can lead to adverse outcomes and heavy economic and social burdens.

Diarrhea is defined as having three or more loose stools within 24 h (3), usually a symptom of gastrointestinal infection. Diarrheal disease causes >500,000 child deaths per year across low- and middle-income countries (4). Children under 5 years old with diarrhea had severe acute malnutrition (14%) and severe stunting (17%) based on the data from the electronic database of Dhaka Hospital from 2019 to 2021 (5). Malaria is a parasitic disease transmitted by mosquitoes and is primarily prevalent in Africa. Malnutrition in children is positively associated with severe malaria incidence rate (6). As a result, each malaria episode increases the risk of malnutrition in children (7). Measles is a viral respiratory infectious disease in children that can lead to serious complications and death in young malnourished children (8). It is estimated that the mortality risk of children with measles is positively correlated with the severity of underweight (9). Respiratory infections and tuberculosis (TB) are among the most common infectious diseases, and nearly 25% people have been infected with TB bacteria worldwide (10). According to statistics, approximately half of patients with TB are malnourished (11). In a word, a strong mutual influence exists between child growth failure and common infectious diseases such as diarrheal diseases, malaria, measles, and respiratory infections and tuberculosis.

However, an assessment of the global burden of common infectious diseases in malnourished children is still lacking. The study aimed to assess the trend of common infectious diseases (diarrheal diseases, malaria, measles, and respiratory infections and tuberculosis) in malnourished children, stratified by age, country, and territory from 1990 to 2021 and to predict future patterns until 2050, which is important for facilitating international aid and WHO decision-making.

## 2 Materials and methods

### 2.1 Data source

Led by the Institute for Health Metrics and Evaluation (IHME), the Global Burden of Diseases, Injuries, and Risk Factors Study (GBD) 2021 serves as an unparalleled platform for assessing the scale of diseases, injuries, and risk factors across various parameters, such as sex, age group, region, and country/territory (UN M49 classification) (12, 13). The estimation of risk factors in GBD 2021 follows a comparative risk assessment framework, encompassing a structured process with the following steps. First, the risk–outcome pairs with credible or convincing evidence were identified. Second, the relative risk in relation to exposure for each specific risk–outcome pair was summarized by incorporating findings from systematic reviews and meta-regression analyses. The relative risks were extracted from primary studies or secondary meta-analyses, both published and unpublished, with adjustments for potential confounding factors by the original researchers. Third, diverse methodologies were employed to assess the exposure levels and distribution across sex, age group, location, and year for each risk factor, utilizing all accessible data sources. Fourth, the theoretical minimum risk exposure level (TMREL) was established as the exposure level linked to the minimum risk, as determined by findings from published trials and cohort studies. Fifth, the population attributable fractions (PAFs) and attributable burden for combined risk factors were estimated, considering the mediating effects, while the RR for each risk–outcome pair, exposure level, and TMREL was used in the modeling of the PAF. The calculation of the PAF follows the subsequent model:

$$\mathrm{PA}F_{\mathrm{aslt}}=\sum_{x=1}^{i}RR_{\mathrm{ast}}(x)P_{\mathrm{aslt}}(x)-1/\sum_{x=1}^{i}RR_{\mathrm{as}}(x)P_{\mathrm{aslt}}(x)$$

where $\mathrm{PA}F_{\mathrm{aslt}}$ is the PAF for disease burden attributable to risk factors for age group *a*, sex *s*, location *l*, and year *t*; $RR_{\mathrm{ast}}$ is the relative risk between exposure level *x* (from 1 to *i*) of risk factors and disease burden for age group *a*, sex *s*, and year *t*; and $P_{\mathrm{aslt}}$ is the proportion of the population exposed to risk factors at level *x* for age group *a*, sex *s*, location *l*, and year (14). Based on the above steps and taking mediating effects into account, the disease burden specifically attributable to risk factors was determined by multiplying the overall burden within each sex, age group, location, and year by the PAF. The data utilized in this investigation were acquired from the GBD database (https://vizhub.healthdata.org/gbd-results/).

### 2.2 Study design

We systematically gathered data on deaths and disability-adjusted life-years (DALYs), along with age-standardized mortality rate (ASMR) and age-standardized DALY rate (ASDR) for patients with diarrheal diseases (ICD-10 codes A00–A09), measles (ICD-10 code B05), malaria (ICD-10 codes B50–B54), and respiratory infections and tuberculosis (CD-10 codes J09–J18, J20–J22, and A15–A19) attributable to child growth failure, child stunting, child underweight and child wasting, which span the period from 1990 to 2021. DALY, which involves the integration of two components, years lived with disability (YLD) and years of life lost (YLL), is a metric used in epidemiology to quantify the overall burden of disease by combining the impacts of mortality and reduced health-related quality of life and reflects the overall impact of a disease on both individual and population health. Stratification followed GBD 2021 protocols: age groups (<1, 1–2, 2–5 years), countries/territories (UN M49 classification), with estimates aggregated using GBD age weights.

### 2.3 Statistical analysis

The estimated annual percentage change (EAPC) is a statistical method commonly employed in epidemiology to quantify the annual rate of change in disease incidence or mortality rates over a specified time period. To determine the EAPC for ASMR and ASDR, a log-linear regression model is applied to the natural logarithms of these rates across the study period. The model is represented by the following equation: ln(ASR) = *β*0 + *β*1 × year + *ε*, where ln(ASR) denotes the natural logarithm of the age-standardized rate, *β*0 is the intercept, *β*1 is the regression slope coefficient that reflects the annual rate of change in the logarithmic transformation of the ASR, and *ε* represents the random error term. The coefficients *β*0 and *β*1 are estimated using ordinary least squares (OLS) regression techniques. Of particular interest is the slope coefficient *β*1, which serves as the basis for calculating the EAPC. This is achieved using the formula EAPC = (exp(*β*1) − 1) × 100, where exp(*β*1) refers to the exponential of the slope, providing the estimated average annual percentage change in the ASR over the study period. The direction and magnitude of the EAPC indicate trends in the ASR: a positive EAPC, with the lower bound of the 95% confidence interval (CI) greater than 0, suggests an upward trend, whereas a negative EAPC, with the upper bound of the 95% confidence interval less than 0, indicates a downward trend.

The Bayesian age–period–cohort (BAPC) model, an extension of the APC model commonly used for analyzing trends in chronic disease incidence and mortality, was employed to project deaths and DALYs, until the year 2050. The parameters included the age groups, period, and birth cohort. The priors included a first-order random walk (RW1) for age effects, period effect, and cohort effect, gamma (1, 0.00005) for variances. Validation was performed by backtesting from 2016 to 2021. After fitting the BAPC model to estimate these effects, we extrapolated them beyond the observed data range to generate future projections with 95% confidence intervals.

To determine the correlation between common infectious diseases (diarrheal diseases, malaria, measles, respiratory infections and tuberculosis) and nutritional deficiencies, we collected and analyzed the incidence rate, deaths, and DALYs of global children under 5 years old through the Global Burden of Disease Study 2021 (GBD 2021), which were analyzed by Pearson correlation analysis and difference Spearman correlation analysis. We reviewed the trends from 1990 to 2021 and analyzed the age groups and geographical factors in 2021 about common infectious diseases among malnourished children under 5 years old. All analyses were conducted using R software (Version 4.3.1).

## 3 Result

### 3.1 Correlation of nutritional deficiencies and common infectious diseases in children under 5 years old

Child growth failure is a major risk factor for several common infectious diseases. Child growth failure contributed 78.91% of the deaths and 77.87% of the DALYs associated with diarrheal diseases for all risk factors. For malaria, child growth failure accounted for 29.71% of deaths and 29.61% of DALYs; 76.85% deaths and 76.64% DALYs for measles were attributable to child growth failure. Child growth failure accounted for 57.06% and 56.19% of deaths and DALYs due to respiratory infections and tuberculosis (Table 1). Moreover, a Pearson correlation analysis of the incidence rate (Supplementary Table 1) revealed that nutritional deficiencies are related to diarrheal diseases (*R* = 0.99, 95% CI: 0.99, 1.00), malaria (*R* = 0.55, 95% CI: 0.24, 0.75), measles (*R* = 0.99, 95% CI: 0.97, 0.99), and respiratory infections and tuberculosis (*R* = 0.77, 95% CI: 0.58, 0.88) with statistical significance. Moreover, using a difference Spearman correlation analysis, we found that nutritional deficiencies were significantly related to diarrheal diseases (*R* = 0.90, 95% CI: 0.79, 0.95) and malaria (*R* = 0.90, 95% CI: 0.79, 0.95) with statistical significance. However, nutritional deficiencies were not significantly associated with malaria and respiratory infections and tuberculosis according to the results of the difference Spearman correlation analysis.

**Table 1:** The total deaths and DALYs of global children under 5 years old with child growth failure with common infectious diseases (diarrheal diseases, malaria, measles, and respiratory infections and tuberculosis) from 1990 to 2021.

Indicator	Group	Diarrheal diseases	Malaria	Measles	Respiratory infections and tuberculosis
Deaths	All risk factors	30,369,913.27	16,304,660.94	9,573,913.40	44,248,427.59
	Child growth failure	23,963,561.60	4,843,597.92	7,357,450.53	25,246,853.58
	Percentage (%)	78.91	29.71	76.85	57.06
DALYs	All patients	2,751,590,117.65	1,461,108,942.32	8,46,424,235.25	3,989,346,921.88
	Child growth failure	2,142,566,674.86	4,32,678,551.18	648,722,281.47	2,241,417,149.06
	Percentage (%)	77.87	29.61	76.64	56.19

### 3.2 Trend of common infectious diseases in children with growth failure

**Figure 1:**
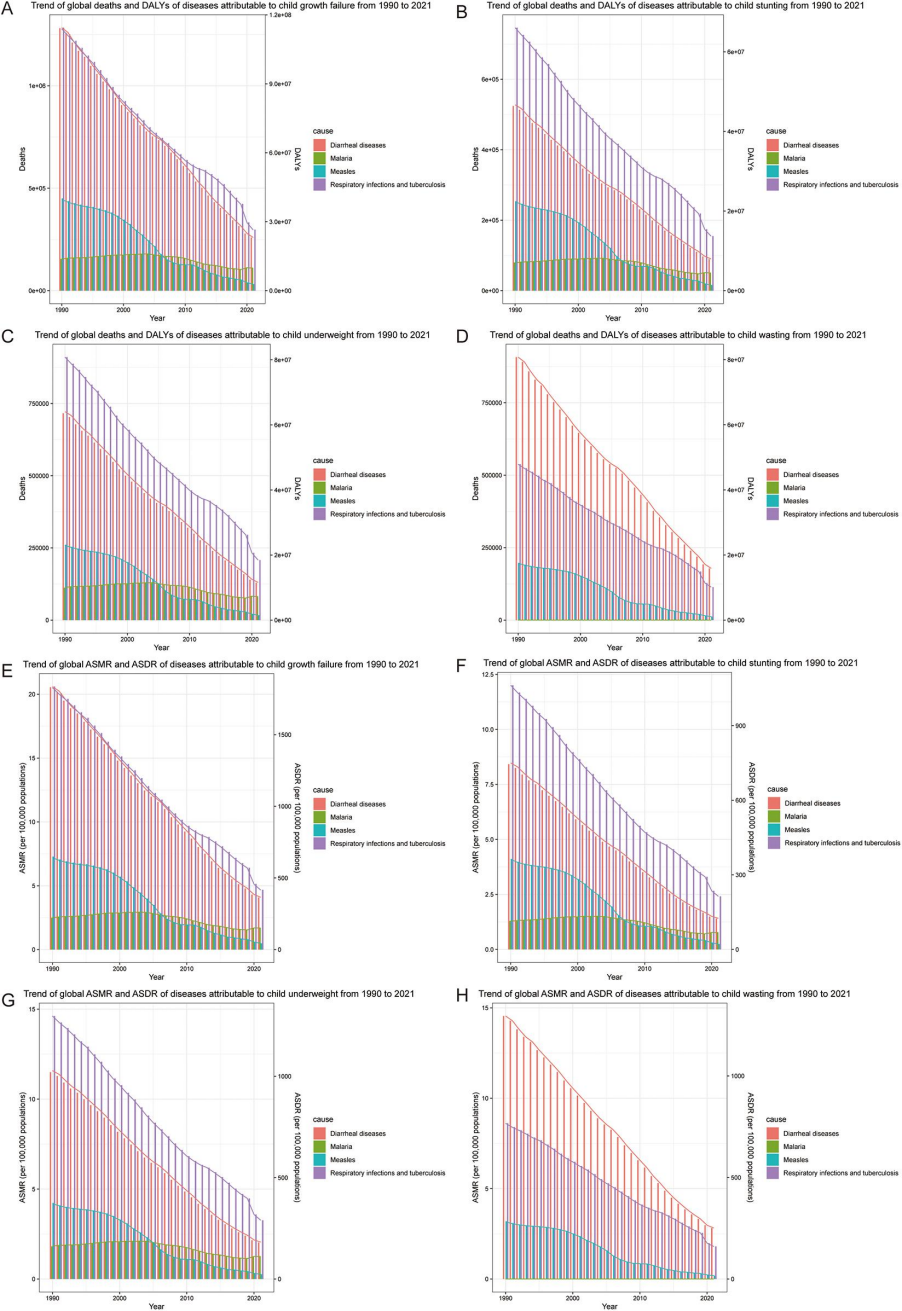
Trend of common infectious diseases in children with growth failure from 1990 to 2019. **(A)** Trend of global deaths and DALYs of diseases attributable to child growth failure. **(B)** Trend of global deaths and DALYs of diseases attributable to child stunting. **(C)** Trend of global deaths and DALYs of diseases attributable to child underweight. **(D)** Trend of global deaths and DALYs of diseases attributable to child wasting. **(E)** Trend of global ASMR and ASDR of diseases attributable to child growth failure. **(F)** Trend of global ASMR and ASDR of diseases attributable to child stunting. **(G)** Trend of global ASMR and ASDR of diseases attributable to child underweight. **(H)** Trend of global ASMR and ASDR of diseases attributable to child wasting.

We reviewed the trends of malnourished children under 5 years old from 1990 to 2021. As shown in Figure 1, the histogram represents deaths or ASMR, and the line graph represents DALYs or ASDR. From 1990 to 2021, deaths and DALYs of diarrheal diseases [EAPC: ASMR = −5.07 (95% CI: −5.37, −4.76), ASDR = −5.06 (95% CI: −5.37, −4.76)], respiratory infections and tuberculosis [EAPC: ASMR = −4.26 (95% CI: −4.50, −4.02), ASDR = −4.25 (95% CI: −4.49, −4.01)] associated with child growth failure tended to decrease. Interestingly, the prevalence of measles markedly decreased in 2006, likely due to the efficient promotion of measles vaccination (15) [EAPC: ASMR = −8.42 (95% CI: −9.08, −7.76), ASDR = −8.42 (95% CI: −9.08, −7.76)]. Notably, the number of deaths and DALYs due to malaria associated with child growth failure has not decreased significantly and has even increased in recent years [EAPC: ASMR = −1.86 (95% CI: −2.33, −1.38), ASDR = −1.85 (95% CI: −2.33, −1.37)] (Table 2). With respect to the different forms of child growth failure (Supplementary Figure S1), child underweight was strongly associated with malaria, measles, and respiratory infections and tuberculosis. Child wasting contributed more to diarrheal diseases.

**Table 2:** Deaths, DALYs, ASMR, ASDR, and EAPC of global children under 5 years old with child growth failure with common infectious diseases (diarrheal diseases, malaria, measles, and respiratory infections and tuberculosis) from 1990 to 2021.

Group	1990				2021				1990–2021 EAPC	
	Deaths	ASMR	DALYs	ASDR	Deaths	ASMR	DALYs	ASDR	ASMR	ASDR
Child growth failure										
Diarrheal diseases	1,280,838.538	20.54950595	114,407,321.6	1,835.380638	259,279.0391	4.081725065	23,178,495.87	364.9646337	−5.07 (−5.37, −4.76)	−5.06 (−5.37, −4.76)
Malaria	154,861.3391	2.504093054	13,823,304.42	223.5021091	109,697.4864	1.687945742	9,806,736.028	150.9444758	−1.86 (−2.33, −1.38)	−1.85 (−2.33, −1.37)
Measles	450,136.7928	7.283716733	39,680,492.44	642.0212213	31,998.64547	0.494716159	2,818,025.713	43.58614879	−8.42 (−9.08, −7.76)	−8.42 (−9.08, −7.76)
Respiratory infections and tuberculosis	1,283,313.792	20.59894978	113,894,015	1,827.892345	298,024.4365	4.680774636	26,442,808.97	415.4865965	−4.26 (−4.50, −4.02)	−4.25 (−4.49, −4.01)
Child stunting										
Diarrheal diseases	524,195.2561	8.419874563	46,688,281.72	749.8498071	89,545.66652	1.403327052	7,979,457.041	125.0886147	−5.6 (−5.95, −5.25)	−5.6 (−5.95, −5.24)
Malaria	79,944.44439	1.295289079	7,017,653.276	113.6901252	50,694.08354	0.775206193	4,442,841.688	67.97022719	−2.23 (−2.78, −1.68)	−2.24 (−2.78, −1.68)
Measles	253,915.1792	4.112566695	22,346,417.71	361.90751	15,975.8124	0.245857543	1,403,951.457	21.61482077	−8.75 (−9.44, −8.05)	−8.75 (−9.44, −8.05)
Respiratory infections and tuberculosis	746,382.3385	12.00652465	66,053,078.04	1,062.377113	154,906.9642	2.412353081	13,691,793.23	213.3322444	−4.65 (−4.91, −4.39)	−4.65 (−4.91, −4.38)
Child underweight										
Diarrheal diseases	716,508.6161	11.49893565	64,052,886.81	1,027.880266	129,466.8433	2.03525777	11,592,673.48	182.2726534	−5.46 (−5.78, −5.13)	−5.45 (−5.77, −5.12)
Malaria	112,245.6432	1.813815066	10,081,089.59	162.8935389	80,123.26956	1.235165521	7,213,487.559	111.2233397	−1.81 (−2.26, −1.36)	−1.8 (−2.25, −1.35)
Measles	261,104.7627	4.225389866	23,000,944.63	372.1871223	17,070.85354	0.263964631	1,502,890.62	23.24857312	−8.72 (−9.41, −8.02)	−8.72 (−9.41, −8.02)
Respiratory infections and tuberculosis	910,647.3659	14.61811007	80,811,442.05	1,297.032769	207,958.4694	3.266127706	18,450,678.71	289.9024172	−4.33 (−4.57, −4.08)	−4.32 (−4.57, −4.08)
Child wasting										
Diarrheal diseases	907,191.1163	14.54761409	80,764,490.64	1,294.988008	178,742.7534	2.818619021	15,916,063.9	251.0559124	−5.13 (−5.46, −4.8)	−5.13 (−5.46, −4.8)
Measles	197,516.31	3.192426583	17,449,750.85	282.0149937	12,144.91731	0.18882085	1,072,525.742	16.68118184	−8.79 (−9.53, −8.04)	−8.79 (−9.52, −8.04)
Respiratory infections and tuberculosis	538,223.5326	8.622192486	47,866,742.37	766.7135518	113,901.7173	1.800118706	10,133,058.88	160.1967155	−4.45 (−4.77, −4.13)	−4.44 (−4.76, −4.13)

### 3.3 Age-specific incidence of common infectious diseases in children with growth failure

**Figure 2:**
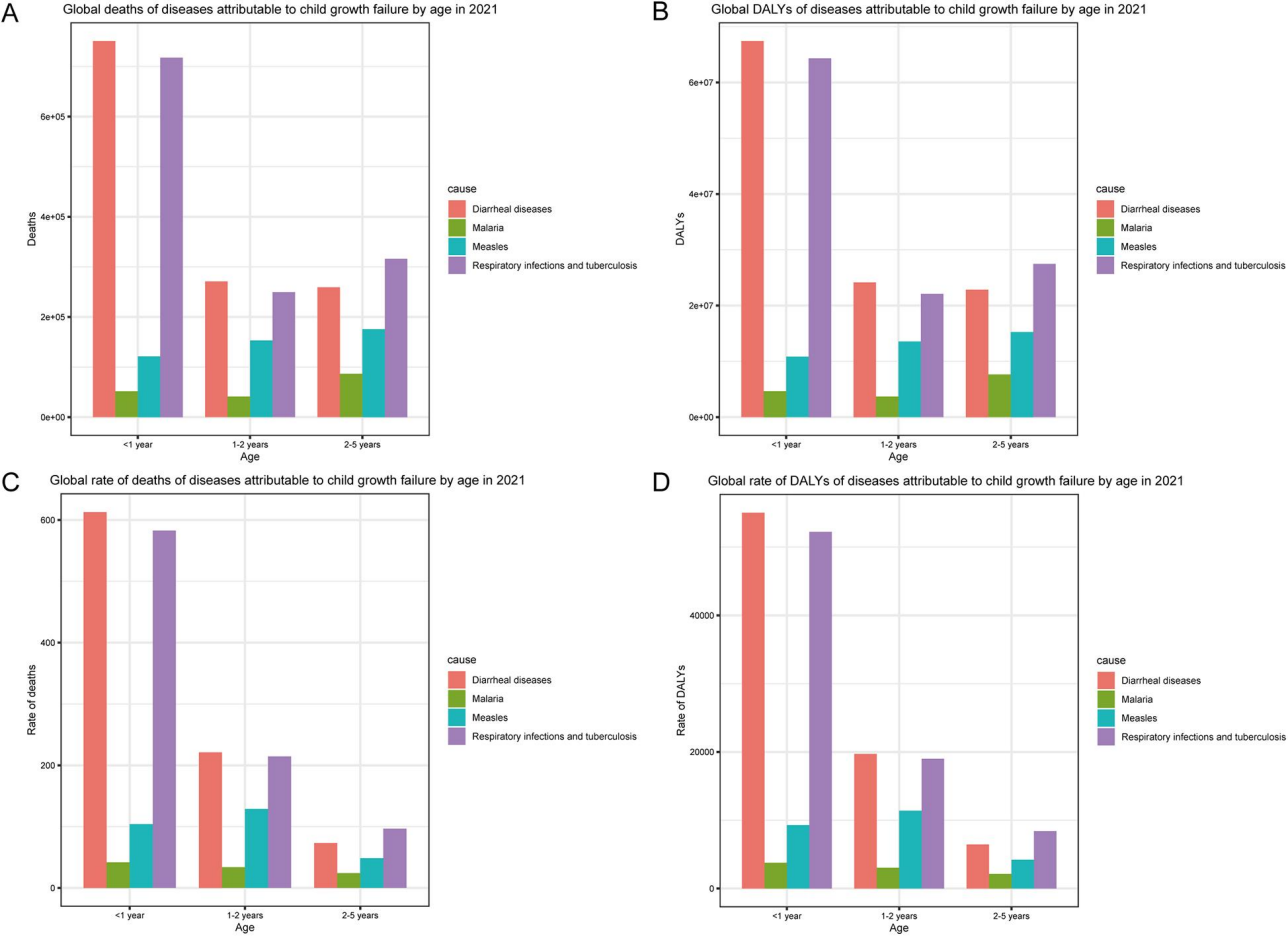
Age-specific incidence of common infectious diseases in children with growth failure in 2021. **(A)** Global deaths of diseases attributable to child growth failure by age in 2021. **(B)** Global DALYs of diseases attributable to child growth failure by age in 2021. **(C)** Global rate of deaths of diseases attributable to child growth failure by age in 2021. **(D)** Global rate of DALYs of diseases attributable to child growth failure by age in 2021.

In terms of age, deaths and DALYs of malnourished children under 5 years old with diarrheal diseases were highest in those aged <1 year, followed by those at 1–2 years and 2–5 years (Figures 2A,B). The deaths and DALYs of children with malaria were highest for those 2–5 years old, second highest for those <1 year old, and lowest for those 1–2 years old. The deaths and DALYs of malnourished children under 5 years old with measles were positively correlated with age. Deaths and DALYs of respiratory infections and tuberculosis mostly occurred in children aged <1 year, followed by those aged 2–5 years and 1–2 years. The rates of death and DALYs among children with diarrheal diseases, malaria, and respiratory infections and tuberculosis were negatively correlated with age (Figures 2C,D). The rates of death and DALYs among malnourished children under 5 years old with measles were highest for those aged 1–2 years, second highest for those aged <1 year, and lowest for those aged 2–5 years. Collectively, according to the 2021 statistics on malnourished children under 5 years old, diarrheal diseases, and respiratory infections and tuberculosis were predominant among infants, whereas malaria and measles were concentrated among toddlers.

### 3.4 Geographical inequality in common infectious diseases in children with growth failure

**Figure 3:**
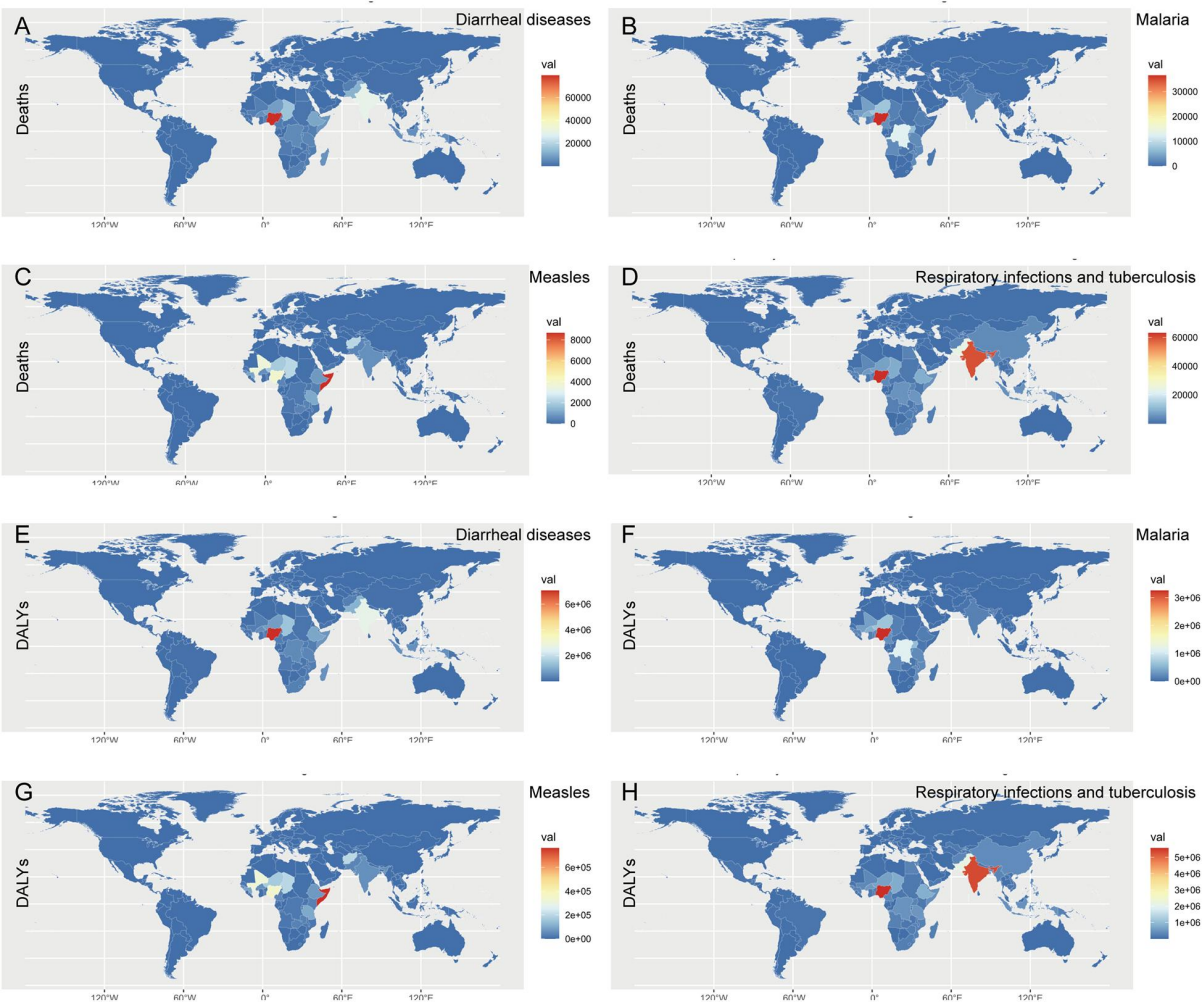
World map of deaths and DALYs of common infectious diseases in children with growth failure in 2021. **(A)** Global deaths map of diarrheal diseases attributable to child growth failure in 2021. **(B)** Global deaths map of malaria attributable to child growth failure in 2021. **(C)** Global deaths map of measles attributable to child growth failure in 2021. **(D)** Global map of respiratory infections and tuberculosis attributable to child growth failure in 2021. **(E)** Global deaths and DALYs map of diarrheal diseases attributable to child growth failure in 2021. **(F)** Global DALYs map of malaria attributable to child growth failure in 2021. **(G)** Global DALYs map of measles attributable to child growth failure in 2021. **(H)** Global DALYs map of respiratory infections and tuberculosis attributable to child growth failure in 2021.

In the map, the majority of diarrheal diseases were prevalent in Nigeria, followed by India, Chad, and Pakistan (Figures 3A,E). Malaria was endemic mainly to Nigeria, the Democratic Republic of the Congo, Niger, and Uganda (Figures 3B,F). Although most countries have achieved the goal of measles elimination, the deaths and DALYs of malnourished children with measles were still high in Somalia and partly in Nigeria and Mali (Figures 3C,G). Respiratory infections and tuberculosis were endemic mainly in Nigeria and India, possibly because of the dense population and poor health care (Figures 3D,H). Finally, diarrheal diseases and malaria were prevalent in Nigeria; measles was endemic in Somalia; and respiratory infections and tuberculosis were widespread in Nigeria and India**.**

### 3.5 Forecasts to 2050: common infectious diseases in children with growth failure

**Figure 4:**
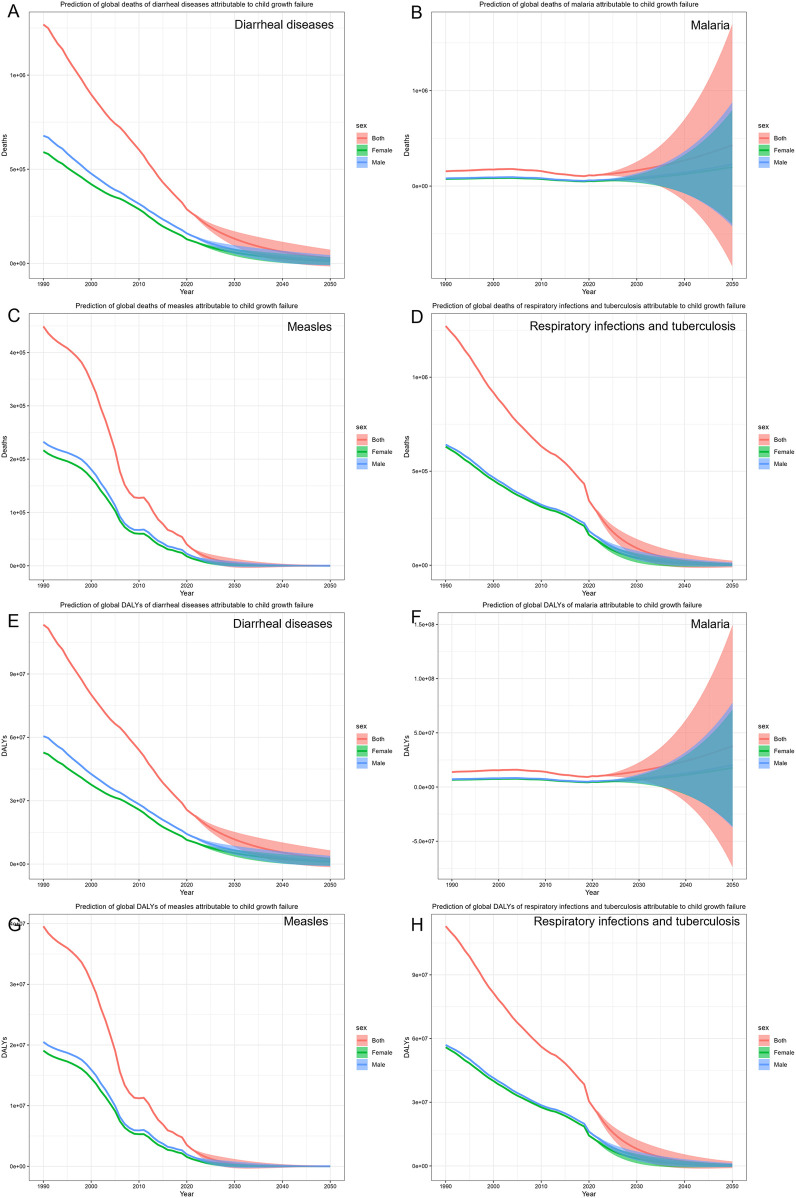
The prediction of common infectious diseases in children with growth failure. **(A)** Prediction of global deaths of diarrheal diseases attributable to child growth failure. **(B)** Prediction of global deaths of malaria attributable to child growth failure. **(C)** Prediction of global deaths of measles attributable to child growth failure. **(D)** Prediction of global deaths of respiratory infections and tuberculosis attributable to child growth failure. **(E)** Prediction of global DALYs of diarrheal diseases attributable to child growth failure. **(F)** Prediction of global DALYs of malaria attributable to child growth failure. **(G)** Prediction of global DALYs of measles attributable to child growth failure. **(H)** Prediction of global DALYs of respiratory infections and tuberculosis attributable to child growth failure.

Further investigations using the BAPC model (Figure 4) revealed that the number of deaths of children with growth failure with diarrheal diseases will decrease from 259,279 (95% CI: 155,943, 358,195) to 28,367 (95% CI: −16,262, 72,998), and the DALYs will decrease from 23,178,495 (95% CI: 13,713,185, 32,003,119) to 2,532,710 (95% CI: −1,456,760, 6,522,180). However, the number of deaths of children with growth failure with malaria will increase from 109,697 (95% CI: −74,808, 353,464) to 428,315 (95% CI: −841,506, 1,698,136), and the number of DALYs will increase from 9,806,736 (95% CI: −6,651,790, 31,438,228) to 37,939,222 (95% CI: −73,887,272, 149,765,717). Deaths and DALYs of measles show a declining trend from 31,998 (95% CI: 15,680, 52,563) to 83 (95% CI: −672, 839) and from 2,818,025 (95% CI: 1,380,526, 4,629,577) to 7,887 (95% CI: −65,298, 81,073), respectively. Deaths and DALYs of respiratory infections and tuberculosis will decrease from 298,024 (95% CI: −212,988, 379,011) to 6,425 (95% CI: −11,471, 24,322) and from 26,442,808 (95% CI: 18,916,574, 33,623,625) to 568,029 (95% CI: −1,019,791, 2,155,851), respectively. Taken together, the BAPC results show that among children with growth failure, the burden of diarrheal diseases, measles, and respiratory infections and tuberculosis is expected to decline gradually in the future. It's worth noting that the malaria burden may increase in the future.

## 4 Discussion

By analyzing the Global Burden of Disease Study 2021 (GBD 2021), this study revealed that child growth failure is closely associated with common infectious diseases, including diarrheal diseases, malaria, measles, and respiratory infections and tuberculosis. With respect to the different forms of child growth failure, child underweight is strongly associated with malaria, measles, and respiratory infections and tuberculosis. Child wasting contributes more to diarrheal diseases. The prevalence of diarrheal diseases, malaria, and respiratory infections and tuberculosis is predominant in children aged <1 year, and the prevalence of measles is predominant at 1–2 years. In terms of regional distribution, diarrheal diseases and malaria are prevalent in Nigeria; measles is endemic in Somalia, and respiratory infections and tuberculosis are widespread in Nigeria and India. Finally, the BAPC results revealed that among children with growth failure, the burden of diarrheal diseases, measles, and respiratory infections and tuberculosis is expected to decline gradually in the future. It's worth noting that the malaria burden may increase in the future.

In our study, we reported that child growth failure contributes to 78.9% deaths and 77.9% DALYs of diarrheal diseases for all risk factors. Undernutrition in children can lead to weakened immunity and increased susceptibility to diarrhea caused by pathogens. Moreover, diarrhea can lead to decreased nutrient absorption and increased energy consumption in children, resulting in undernutrition. Our research revealed a correlation between childhood undernutrition and diarrhea through data analysis. Our research found that for malaria, child growth failure accounts for 29.7% of deaths and 29.6% of DALYs. A cohort study from Asembo Bay, with a sample of 1,182 children, revealed that undernutrition in children is associated with afebrile malaria (16). Moreover, recent studies have shown that undernutrition among children is positively related to severe malaria (6). Our research indicated that 76.8% deaths and 76.6% DALYs among children with measles are attributable to child growth failure. Undernutrition can lead to secondary immunodeficiencies, especially in children (17). Severe measles, including severe pneumonia and acute disseminated encephalomyelitis, is mainly seen in children under 5 years old with poor nutritional status (18), especially those deficient in vitamin A (19). Our research revealed that child growth failure accounts for 57.1% and 56.2% of deaths and DALYs due to respiratory infections and tuberculosis, respectively. Previous studies have suggested that undernutrition can reduce the levels of protective cytokines (20), chemokines (21), B cell count, and natural killer (NK) cell count (22) in tuberculosis patients. Moreover, undernutrition in patients with TB increases the risks of lung cavitation (23), drug resistance (24), and poor prognosis (25), which is consistent with our results. In a word, varying degrees of interaction exist between child growth failure and common infectious diseases such as diarrhea, malaria, measles, respiratory tract infections, and tuberculosis.

Common infectious diseases are more prevalent in different age groups. The rate of deaths and DALYs among malnourished children with diarrheal diseases, and respiratory infections and tuberculosis was significantly negatively correlated with age. Children <1 year have fewer total body fluids, and children's water turnover rate is negatively correlated with age (26), which makes them more susceptible to frequent diarrhea-associated water–electrolyte disturbances, leading to a poor prognosis. The immature immune system of infants is prone to disease, which is further exacerbated by malnutrition (27). The malnourished infants cannot effectively eliminate malaria parasites and are prone to severe malaria (28). Bacillus Calmette–Guérin (BCG) cannot completely prevent the occurrence of tuberculosis. The malnourished infants are prone to severe tuberculosis, such as tuberculous meningitis and acute miliary pulmonary tuberculosis (29). The rate of deaths and DALYs among malnourished children under 5 years old with measles was highest among those aged 1–2 years. Measles is a viral infection. Infants with maternal antibodies or with a vaccination are less likely to be infected with measles than toddlers are (30). It is important to focus on preventing common infectious diseases among malnourished children according to different age groups.

Moreover, we collected geographical distribution data on common infectious diseases among malnourished children under 5 years old in 2021 across countries and territories. Our research revealed that diarrheal diseases and malaria are most prevalent in Nigeria worldwide. Nigeria has the largest population in Africa. Its population is projected to increase by 58% by 2050, and the under-five mortality rate is the second highest in the world (31). Our research has demonstrated the close relationship between child undernutrition and common infectious diseases. It's very important to improve child undernutrition. Some studies suggest that the government should focus on maternal support during pregnancy for women of childbearing age, pregnant women, and children under 24 months of age and nutritional supplementation for those who are food insecure (32). Our research indicated that among malnourished children under 5 years old worldwide, measles is most endemic in Somalia. The complex political environment in Somalia has led to the displacement of many people, which has resulted in poor sanitation conditions and outbreaks of measles (33). Measles vaccination significantly decreases the prevalence of measles, even with only a single dose of the vaccine (34). The implementation of measles vaccination is very valuable and can be appropriately rewarded with economic incentives. Our research revealed that among malnourished children under 5 years old, respiratory infections and tuberculosis are most prevalent in Nigeria and India. According to the WHO report, 28% of new tuberculosis cases and 38% of global tuberculosis deaths each year occur in India (35).The number of incident tuberculosis cases attributable to selected risk factors and undernourishment tops the list of risk factors. The interaction between child undernutrition and respiratory infections and tuberculosis should be taken seriously.

Furthermore, we forecast that the deaths and DALYs burden of malaria may rise in the future among malnourished children under 5 years old by the BAPC model. The rise of malaria is highly noteworthy in the context of other common infectious diseases obviously decreasing in the future. The government should invest more funds in insecticide-treated nets (ITNs) and indoor residual spraying (IRS) to prevent the spread of malaria. In addition, preventive chemotherapy for malnourished children should be provided.

This study still has some limitations. First, the GBD database relies heavily on data reporting from individual countries. However, low-income countries often lack well-established disease surveillance networks, which may result in the absence of certain data. Second, the GBD data consists of point estimates and the CI. When we conduct analyses, we can only use point estimates for calculations, which may lead to a decrease in credibility. Third, the CI of BAPC prediction for malaria research is relatively wide. The total number of child growth failure (CGF)-attributed malaria fatalities is much smaller than that for other diseases; thus, Poisson noise increases exponentially as the event count decreases. Malaria transmission is highly sensitive to climate and control interventions such as ITN distribution and chemoprevention, and frequent data updates are needed to capture its dynamic changes.

## 5 Conclusions

In summary, this study emphasizes the burden of common infectious diseases (diarrheal diseases, malaria, measles, and respiratory infections and tuberculosis) in children with growth failure and facilitates international aid and WHO decision-making targeting countries and age groups.

## Data Availability

Publicly available datasets were analyzed in this study and can be found here: https://vizhub.healthdata.org/gbd-results/.

## Supplementary material

Supplemental Figure 1 Trend of different forms of growth failure attributable to common infectious diseases from 1990 to 2019. (A) Trend of global deaths and DALYs of diseases attributable to diarrheal diseases. (B) Trend of global deaths and DALYs of diseases attributable to malaria. (C) Trend of global deaths and DALYs of diseases attributable to measles. (D) Trend of global deaths and DALYs of diseases attributable to respiratory infections and tuberculosis. (E) Trend of global ASMR and ASDR of diseases attributable to diarrheal diseases. (F) Trend of global ASMR and ASDR of diseases attributable to malaria. (G) Trend of global ASMR and ASDR of diseases attributable to measles. (H) Trend of global ASMR and ASDR of diseases attributable to respiratory infections and tuberculosis.
