# Supplementary figures and images for: Burden of common infectious diseases in children with growth failure from 1990 to 2021: analysis of the Global Burden of Disease Study
Source: Front Pediatr. 2025 Nov 7;13:1648964. doi: 10.3389/fped.2025.1648964 (PMC12634535; doi:10.3389/fped.2025.1648964)

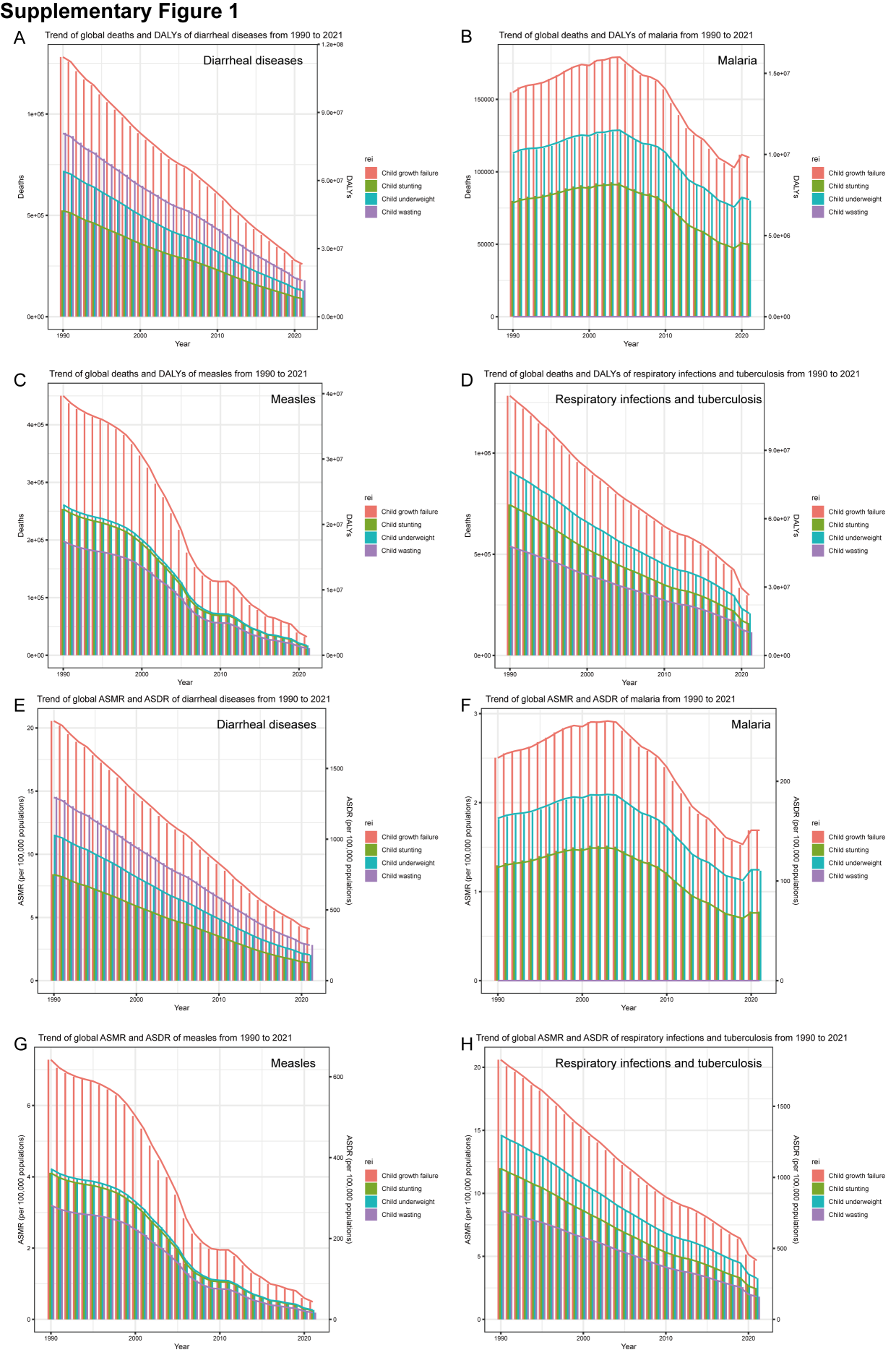

Supplement: Supplemental Figure 1 — Trend of different forms of growth failure attributable to common infectious diseases from 1990 to 2019. (A) Trend of global deaths and DALYs of diseases attributable to diarrheal diseases. (B) Trend of global deaths and DALYs of diseases attributable to malaria. (C) Trend of global deaths and DALYs of diseases attributable to measles. (D) Trend of global deaths and DALYs of diseases attributable to respiratory infections and tuberculosis. (E) Trend of global ASMR and ASDR of diseases attributable to diarrheal diseases. (F) Trend of global ASMR and ASDR of diseases attributable to malaria. (G) Trend of global ASMR and ASDR of diseases attributable to measles. (H) Trend of global ASMR and ASDR of diseases attributable to respiratory infections and tuberculosis. [file Image1.tiff]
