# Supplementary material for: Burden of common infectious diseases in children with growth failure from 1990 to 2021: analysis of the Global Burden of Disease Study
Source: Front Pediatr. 2025 Nov 7;13:1648964. doi: 10.3389/fped.2025.1648964 (PMC12634535; doi:10.3389/fped.2025.1648964)
Supplement: Supplementary file 2 [file Table1.docx]

##### Supplemental Table 1. The correlation of incidence rate between nutritional deficiencies and common infectious diseases (diarrheal diseases, malaria, measles, respiratory infections and tuberculosis) among children under 5 years from 1990 to 2021.

| Incidence rate | Nutritional deficiencies | Diarrheal diseases | Malaria | Measles | Respiratory infections and tuberculosis |
| --- | --- | --- | --- | --- | --- |
| 1990 | 41708.79 | 190036.82 | 14185.63 | 8611.06 | 360819.06 |
| 1991 | 41057.13 | 185125.93 | 14303.34 | 8390.41 | 360602.19 |
| 1992 | 40497.20 | 180693.45 | 14514.15 | 8257.48 | 360442.44 |
| 1993 | 40049.60 | 177000.00 | 14749.22 | 8203.94 | 360337.85 |
| 1994 | 39701.55 | 174160.09 | 15064.30 | 8198.97 | 360297.72 |
| 1995 | 39431.53 | 172254.50 | 15474.61 | 8226.51 | 360261.90 |
| 1996 | 39131.72 | 170693.43 | 15896.47 | 8260.37 | 359558.83 |
| 1997 | 38755.46 | 168912.55 | 16367.61 | 8254.68 | 357914.31 |
| 1998 | 38324.63 | 167014.91 | 16727.53 | 8188.23 | 355879.78 |
| 1999 | 37872.61 | 165127.22 | 17052.34 | 7919.85 | 354029.10 |
| 2000 | 37424.52 | 163370.51 | 16856.88 | 7742.49 | 352934.90 |
| 2001 | 36880.01 | 161410.10 | 17287.36 | 7485.57 | 352182.95 |
| 2002 | 36177.20 | 158943.84 | 17049.58 | 7169.17 | 351006.88 |
| 2003 | 35391.21 | 156173.17 | 17188.25 | 6808.78 | 349520.65 |
| 2004 | 34582.48 | 153304.42 | 17265.91 | 6520.86 | 347906.29 |
| 2005 | 33815.39 | 150578.40 | 17009.07 | 5939.52 | 346371.01 |
| 2006 | 33028.45 | 147476.82 | 16744.19 | 5248.56 | 343848.64 |
| 2007 | 32123.13 | 143535.91 | 16540.61 | 4564.35 | 339812.62 |
| 2008 | 31132.28 | 139120.07 | 16350.04 | 4148.05 | 335328.82 |
| 2009 | 30119.60 | 134637.65 | 16216.72 | 3969.67 | 331436.23 |
| 2010 | 29156.83 | 130524.67 | 15888.25 | 3988.50 | 329173.64 |
| 2011 | 28198.34 | 125677.71 | 15424.57 | 3875.79 | 328464.97 |
| 2012 | 27169.89 | 119112.38 | 14793.38 | 3483.17 | 328433.93 |
| 2013 | 26101.73 | 111543.43 | 14269.32 | 2960.21 | 328869.23 |
| 2014 | 25030.85 | 103772.84 | 13789.37 | 2510.27 | 329423.57 |
| 2015 | 23972.69 | 96479.74 | 13540.36 | 2237.77 | 329656.66 |
| 2016 | 22685.11 | 88410.05 | 13428.17 | 2088.97 | 329824.09 |
| 2017 | 21181.42 | 79304.83 | 13449.53 | 2106.89 | 330336.06 |
| 2018 | 19818.07 | 71157.29 | 13514.54 | 1763.94 | 330964.09 |
| 2019 | 18900.39 | 65732.88 | 13777.57 | 1413.59 | 331412.61 |
| 2020 | 18485.67 | 63199.47 | 14323.86 | 947.26 | 347162.59 |
| 2021 | 18240.73 | 59677.27 | 14631.95 | 619.94 | 352318.01 |
| Pearson correlation coefficient R |  | 0.99 | 0.55 | 0.99 | 0.77 |
| 95% CI |  | 0.99-1.00 | 0.24-0.75 | 0.97-0.99 | 0.58-0.88 |
| P-value |  | < 0.0001 | 0.0012 | < 0.0001 | < 0.0001 |
| Difference Spearman correlation coefficient R |  | 0.90 | 0.90 | -0.15 | -0.13 |
| 95% CI |  | 0.79-0.95 | 0.79-0.95 | -0.52-0.23 | -0.40-0.18 |
| P-value |  | < 0.0001 | < 0.0001 | 0.435 | 0.497 |
